# Identification of Genes Associated with Reproduction in the Mud Crab (*Scylla olivacea*) and Their Differential Expression following Serotonin Stimulation

**DOI:** 10.1371/journal.pone.0115867

**Published:** 2014-12-26

**Authors:** Napamanee Kornthong, Scott F. Cummins, Charoonroj Chotwiwatthanakun, Kanjana Khornchatri, Attakorn Engsusophon, Peter J. Hanna, Prasert Sobhon

**Affiliations:** 1 Chulabhorn International College of Medicine, Thammasat University, Pathumthani, 12121, Thailand; 2 Department of Anatomy, Faculty of Science, Mahidol University, Rama VI Road, Ratchathewi, Bangkok, 10400, Thailand; 3 Faculty of Science, Health, Education and Engineering, University of the Sunshine Coast, Maroochydore, Queensland, 4558, Australia; 4 Mahidol University, Nakhonsawan Campus, Nakhonsawan, 60130, Thailand; 5 Pro Vice-Chancellor's Office, Faculty of Science, Engineering and Built Environment, Deakin University, Locked Bag 20000, Geelong, Victoria, 3220, Australia; Uppsala University, Sweden

## Abstract

The central nervous system (CNS) is often intimately involved in reproduction control and is therefore a target organ for transcriptomic investigations to identify reproduction-associated genes. In this study, 454 transcriptome sequencing was performed on pooled brain and ventral nerve cord of the female mud crab (*Scylla olivacea*) following serotonin injection (5 µg/g BW). A total of 197,468 sequence reads was obtained with an average length of 828 bp. Approximately 38.7% of 2,183 isotigs matched with significant similarity (E value < 1e^−4^) to sequences within the Genbank non-redundant (nr) database, with most significant matches being to crustacean and insect sequences. Approximately 32 putative neuropeptide genes were identified from nonmatching blast sequences. In addition, we identified full-length transcripts for crustacean reproductive-related genes, namely farnesoic acid o-methyltransferase (*FAMeT*), estrogen sulfotransferase (*ESULT*) and prostaglandin F synthase (*PGFS*). Following serotonin injection, which would normally initiate reproductive processes, we found up-regulation of *FAMeT*, *ESULT* and *PGFS* expression in the female CNS and ovary. Our data here provides an invaluable new resource for understanding the molecular role of the CNS on reproduction in *S. olivacea*.

## Introduction

Among all crustaceans, the mud crab *Scylla olivacea*, is one of the most important species for global aquatic production due to the quality and taste of their eggs and meat [1]. As an outcome of the high economic value, there has been focus on how to increase quantity and quality. Unfortunately, there is currently little molecular knowledge of the genes and proteins involved in reproduction of this species, and therefore it had become of paramount importance to undertake a transcriptomic investigation to identify these within relevant tissues.

The central nervous system (CNS) is a crucial tissue for the study of neuroanatomical, neurochemical and neurophysiological aspects of biological functioning in crustaceans. In *S. olivacea*, the CNS neuroanatomy has recently been described [2]. Within the CNS, neuronal clusters and fibers appear in distinct regions that likely provide different functions to allow for a complete neuronal circuit. The ultimate functional importance of each neuronal cluster during adult neurogenesis remains unclear, despite the findings that in the adult crayfish brain neurogenesis takes place in the optic neuropils and neuronal cluster 10 [3]. Immunocytochemistry has also revealed the existence of several neurosecretory cells in the CNS that contain neurotransmitters and neurohormone-like proteins involved in reproduction [4]–[6].

A variety of neurotransmitters and neurohormones have been identified in the CNS and appear to control physiological and behavioral functions in crustaceans [7], [8]. For example, serotonin (5-hydroxytryptophan, or 5-HT) is one neurotransmitter that has been extensively studied and found to control various functions, including aggression, as well as increased growth rate and gonad maturation in crabs and prawns [9]–[11]. This is supported by the observed presence of 5-HT in CNS and gonad [6], [10]. In the CNS of *S. olivacea*, 5-HT is distributed throughout, particularly in several neuronal clusters and fibers of the brain (Khornchatri et al., unpublished), although there has been no evidence to date that 5-HT controls reproduction. However, 5-HT injection can up-regulate the expression of the red pigment concentrating hormone [7], a hormone that is important for body color change and hypothesized to be involved in the regulation of reproduction hormones.

Overall, the study of crustacean reproduction genes is limited due to the lack of genome databases. *Daphnia pulex* (water flea) is currently among the best-studied crustaceans for evolutionary environmental genomics due to the availability of its genome sequence [12]. It provides a basis for comparative analyses with genes identified in other crustaceans. Moreover, transcriptomic data from other crustaceans has been expanding rapidly through the relative ease of sequencing transcripts [13]–[18]. In this study, we investigated a neural transcriptome (pooled brain and ventral nerve cord) of *S. olivacea*, following 5-HT induction to assess the presence of reproductive-related genes and to understand putative reproduction control mechanisms. We have identified numerous candidate genes and gene families involved in reproduction in the female CNS and ovary, as well as putative neuropeptides of unknown function.

## Results and Discussion

### Roche 454 GS-FLX sequencing and contig assembly

Messenger RNAs purified from the brain and ventral nerve cord of 5-HT-injected female *S. olivacea* were sequenced using the 454 GS-FLX platform for *de novo* assembly. A total of 197,468 sequence reads were generated that were further assembled into 2,183 isotigs, with a median sequence length of 828 bp for each isotig. A total of 1,236 contigs were greater than 500 bp, with the largest contig at 11,940 bp, and many providing full-length open reading frames (ORFs). A representation of the full-length open reading frames (ORFs) is shown in **S1 Table**. A further 44,721 singletons were identified. All sequence reads are available in the Gene Expression Omnibus at the National Center for Biotechnology Information (NCBI) database with the accession number SRR1213089.

This transcriptome data provides the first in-depth identification of the genes expressed in the *S. olivacea* CNS, enabling a platform for further investigations, such as homology searches, gene ontology (GO) annotation prediction, enzymatic pathway prediction, neuropeptide prediction and gene and protein expression analysis.

### Comparative analyses of *S. olivacea* CNS gene transcripts

**Figure 1:**
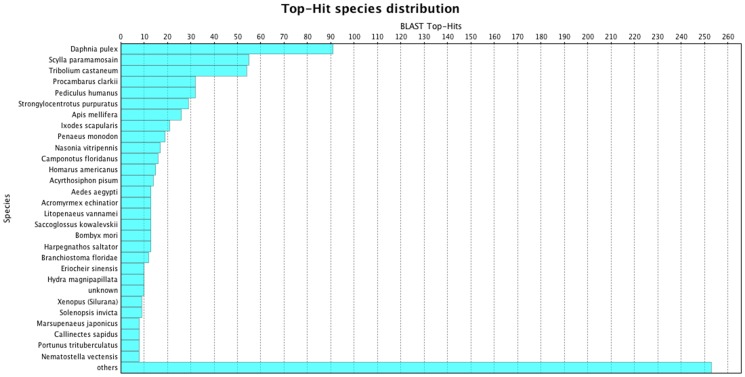
Blastx top hits (30 species) using *S. olivacea* CNS isotigs (E-value cut-off 1e^−4^). Distribution of known species is primarily crustacean and other arthropods.

Neural *S. olivacea* gene transcripts were submitted for similarity to the GenBank non-redundant (nr) database by the blastx searching tool. Of 2,183 *S. olivacea* isotigs that had matches, the majority (844 isotigs, 38.66%) matched to proteins with a known function in crustaceans or arthropods (E-value > 1e^−4^) present within the GenBank non-redundant (nr) database **(** **Fig. 1** **)**, primarily due to a previous transcriptome study of *Macrobrachium rosenbergii* [13]. Some sequences were also matched to hypothetical proteins of unknown function. After redundant and ribosomal proteins were excluded, putative genes were identified and analyzed further. *D. pulex* is most commonly represented in blastx searches, as well as some penaeid shrimp, crabs, crayfish, as well as other arthropods. Approximately 1,339 isotigs (61.34%) did not match with any genes in the NCBI database, which exemplifies the lack of gene sequences for *S. olivacea* and other decapod crustaceans in available online public databases.

### Gene Ontology assignments and KEGG analysis

**Figure 2:**
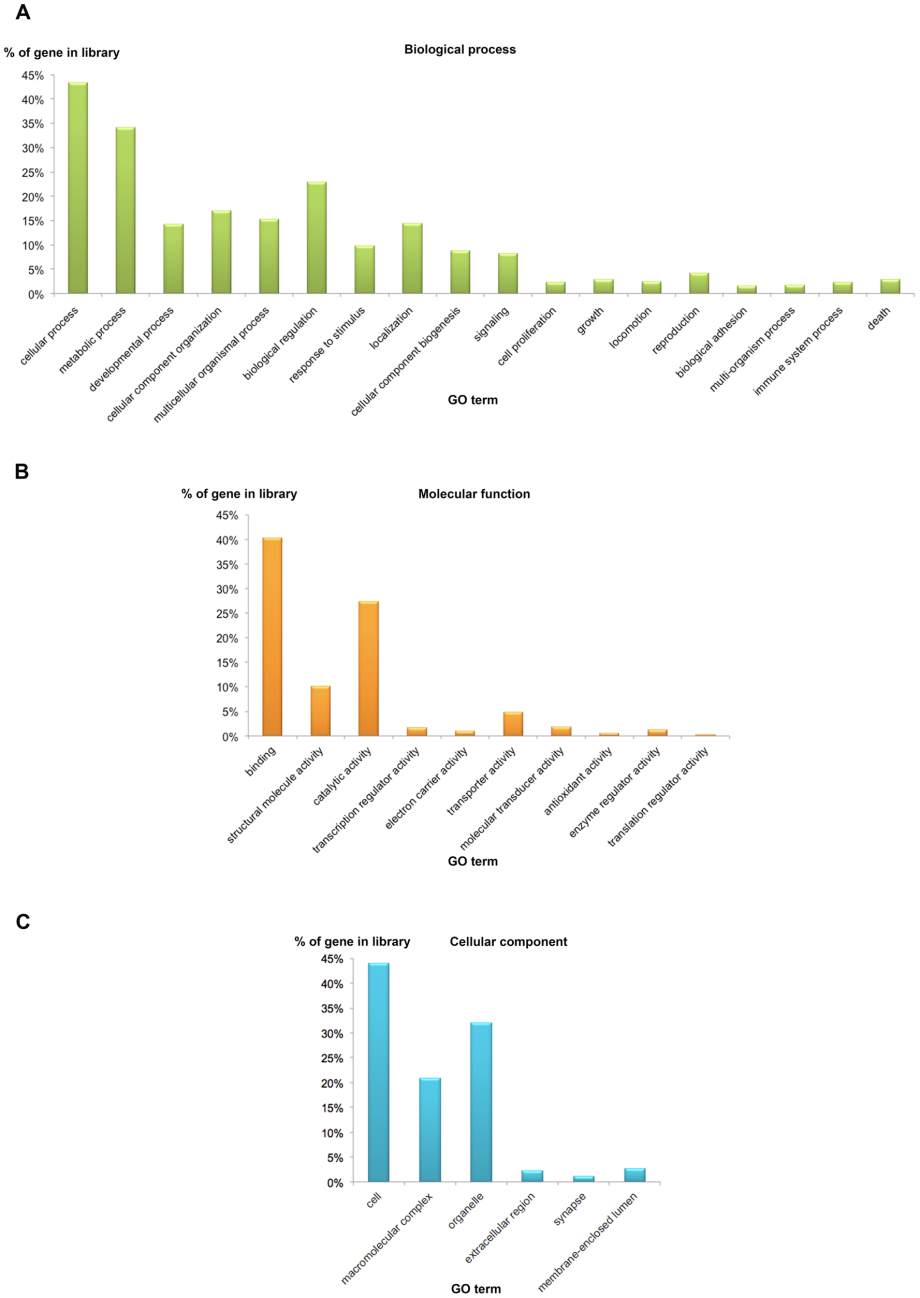
Gene ontology (GO) annotations (level 2 terms) for the transcriptomic sequences of *S. olivacea*. (A) biological process, (B) molecular function, and (C) cellular component.

A total of 844 isotigs that matched to known sequences were assigned for gene ontology (GO) analysis using the Blast2GO program, based on matches with sequences in which the function was already known in the GenBank non-redundant (nr) protein database of NCBI **(** **Fig. 2** **)**. Among these, 766 isotigs were annotated successfully with confident matches. Several of the sequences were assigned to one or more ontologies due to the similarity of sequences with previous known functions.

Coding sequences were assigned to biological processes (414 sequences; 49.05%). Primarily cellular process (367; 43.4%), metabolic process (288; 34.12%), and biological regulation (193; 5.01%) in level 2 terms were the most abundant terms in the biological processes **(** **Fig. 2A** **)**. Approximately 455 sequences (53.9%) could be classified according to molecular function, with the most abundant groupings to binding (340; 40.28%), structural molecular activity (85; 10.07%) and catalytic activity (231; 27.3%) in level 2 terms **(** **Fig. 2B** **)**. GO analysis assigned 382 sequences (44.07%) to a potential cellular component, with the most abundant including cell (372; 44.07%), macromolecular complex (176; 20.85%), and organelle (271; 32.1%) in level 2 terms **(** **Fig. 2C** **)**. Approximately 19 sequences (2.25%) were predicted to function in the extracellular region, which may comprise putative neuropeptides. For the *S. olivacea* transcriptome, potential functions conformed with GO annotation of other crustaceans expressed sequence tags [19]–[22].

Isotigs were mapped to the KEGG database to define metabolic pathways. According to these results, 844 isotigs could be mapped into 59 different metabolic pathways. The *S. olivacea* CNS transcripts were most prominent within metabolic pathways of oxidative phosphorylation, nitrogen metabolism, glycolysis/gluconeogenesis, and purine metabolism **(** **S2 Table** **)**. These KEGG analyses provide a helpful insight for future investigations focusing on gene function.

### Reproduction-associated genes present in the *S. olivacea* CNS and ovary

In this study, we investigated the CNS (pooled brain and ventral nerve cord) transcriptome of *S. olivacea* following 5-HT induction. To date, few genes have been well characterized in *S. olivacea* with known reproductive-associated function in contrast to some other crustaceans or arthropods [7]. In the current study, we obtained full-length transcripts for reproduction-associated genes. However, a gonadotropin-releasing hormone (GnRH) family neuropeptide transcript was not found in this transcriptome [7], [23], perhaps due to its low expression level. Of those reproduction-associated genes identified, the deduced amino acid sequence of each encoded protein was shown to have some similarity with other species.

#### *Farnesoic acid o-methyltransferase (FAMeT)*

**Figure 3:**
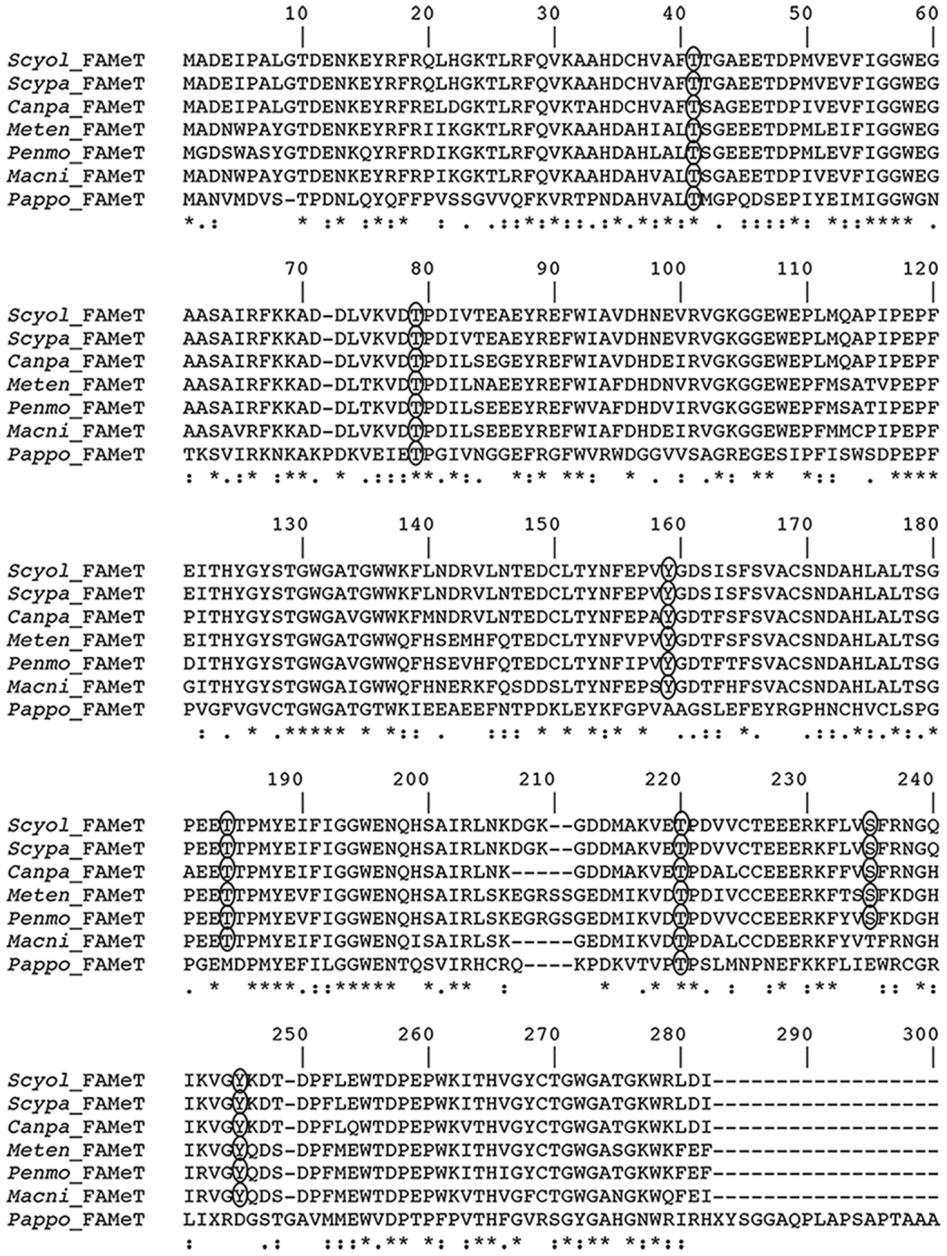
Alignment of amino acid sequences of farnesoic acid o-methyltransferase (*FAMeT*) of *S. olivacea* with known arthropod species. Gaps (-) included to allow for alignment. “*”: identical amino acids; “:”: conserved substitutions (same group); “.”: semi-conserved substitution (similar shapes). Circles indicating phosphorylation site prediction on protein sequences. (*Scyol*, *Scylla olivacea*) orange mud crab; (*Scypa*, *Scylla paramamosain*), green mud crab (Accession number ADK32330); (*Canpa*, *Cancer pagurus*), edible crab (AAR00732); (*Meten*, *Metapenaeus ensis*), sand shrimp (AAK28535); (*Penmo*, *Penaeus monodon*), black tiger shrimp (AAX24112); (*Macni*, *Macrobrachium nipponense*), freshwater prawn (AFA26604); and (*Pappo*, *Papilio polytes*), butterfly (BAM20323).

FAMeT has been reported as an important enzyme required for the synthesis of methyl farnesoate (MF), a crustacean reproductive hormone that is structurally similar to insect juvenile hormone [24], [25]. A sequence for *Scyol-FAMeT* was identified in the *S. olivacea* CNS and ovary. FAMeT plays a central role in catalyzing methylation of farnesoic acid (FA) to MF with a rate limiting step enzyme [26]. MF has been extensively linked to molting, development and general protein synthesis in crustaceans and other arthropods [27]–[29]. Also, MF is associated with reproduction where it has been strongly suggested to stimulate ovarian maturation in *Procambarus clarkii, Macrobrachium malcolmsonii, Oziotelphusa senex senex, Libinia emarginata* [30]–[34]. In the current study, we show the full-length protein encoded within the *Scyol-FAMeT* transcript (**S1 Fig** **.)**, and in comparison with other crustaceans **(** **Fig. 3** **)**. *S. olivacea FAMeT* shows identical sequence similarity to *Scylla paramamosain* (100%), also high similarity to *Cancer pagurus* (90%), *Metapenaeus ensis* (79%), *Macrobachium nipponense* (79%), and *Penaeus monodon* (77%), but relatively less to *Papilio polytes* (37%). Additional analysis of *FAMeT* protein sequence, on possible phosphorylation sites (score >8) at serine, threonine and tyrosine side chains was also shown **(** **Fig. 3** **)**.

**Figure 4:**
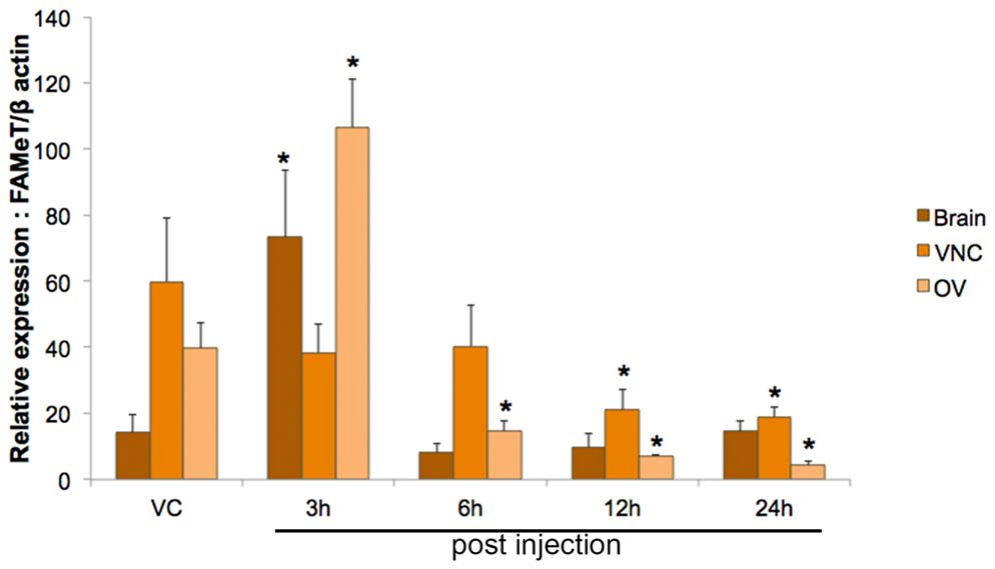
Effect of priming 5-HT on relative *FAMeT* gene expression level in *S. olivacea* using quantitative RT-PCR. *FAMeT* mRNA expression levels in the brain, VNC and ovary (n = 12) were determined at 3, 6, 12 and 24 h post 5-HT injection. Data were normalized against *β*-actin. Asterisks indicate significant differences (*p<*0.05) with respect to the control (vehicle-injected) group. Abbreviations: farnesoic acid o-methyltransferase (FAMeT), ventral nerve cord (VNC), ovary (OV), and vehicle control (VC).

We investigated the differential expression of *FAMeT* following 5-HT induction using quantitative real-time PCR. Mature female mud crabs were injected with 5-HT (5 µg/g BW) over a period of 3 h which has previously been shown to stimulate reproduction [7], then compared expression profiles to with control vehicle (saline). The *Scyol*-*β*-actin was used to normalize target gene expression to enable assessment of relative changes in gene expressions in the brain, ventral nerve cord (VNC) and ovary. Our results indicate that following 5-HT injection, *Scyol-FAMeT* expression in the brain and ovary increased significantly (*p*<0.05) at 3 h post-injection and subsequently decreased at 6, 12 and 24 h post-injection. Meanwhile, VNC *Scyol-FAMeT* showed a significant decrease (*p*<0.05) in 5-HT-injected animals at 12 and 24 h post-injection **(** **Fig. 4** **)**. This result concurs with a previous report in the shrimp, *Fenneropenaeus merguiensis*, which indicated that hemolymph MF levels increased after injection with 5-HT [35]. Indeed, some neuropeptides have been reported to regulate MF production either positively or negatively; and this includes the red pigment concentration hormone, which stimulates the mandibular organ (MO) to synthesize MF. In contrast, MF synthesis is inhibited by pigment-dispersing hormone, perhaps through its inhibition of FAMeT activity [36]. Hence, the accumulating data imply that MF is involved in reproduction, either directly or indirectly. Although FAMeT has been reported to have biological activity only on the MO in *H. americanus* [37], we suggest that 5-HT might have some effect on the brain and ovary in the expression of *FAMeT* mRNA in *S. olivacea*. A relationship with 5-HT and FAMeT has never been reported in a crustacean, but it is known that hemolymph MF increases after 5-HT injection [35]. Our suggestion needs to be explored further and confirmed in other species of decapod crustaceans.

#### *Estrogen sulfotransferase (ESULT)*

**Figure 5:**
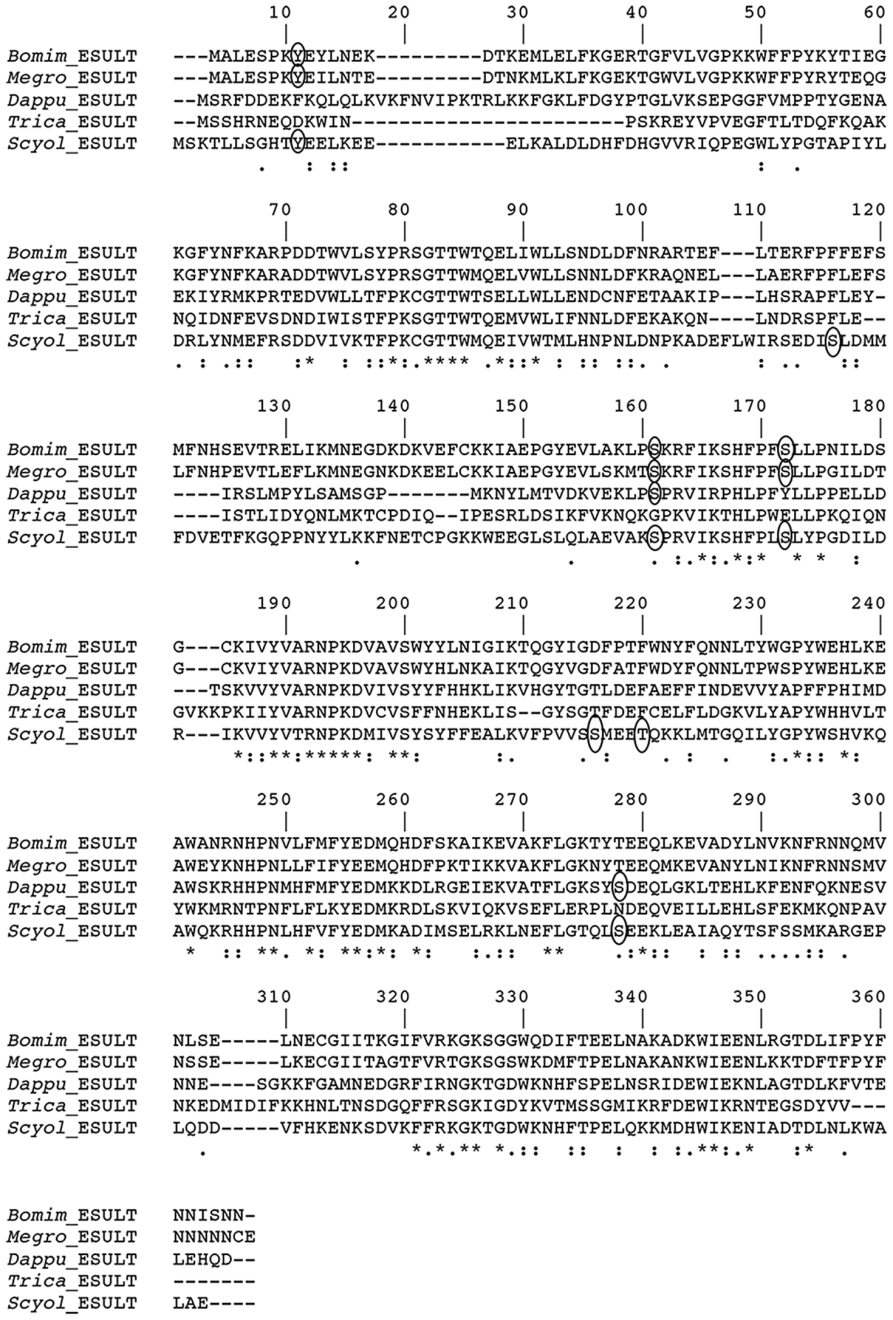
Alignment of *S. olivacea* amino acid sequence of estrogen sulfotransferase (*ESULT*) with similar sequence from other species. Gaps (-) included to allow for alignment. “*”: identical amino acids; “:”: conserved substitutions (same group); “.”: semi-conserved substitution (similar shapes). Circles indicating phosphorylation site prediction on protein sequences. (*Scyol*, *Scylla olivacea*), orange mud crab; (*Trica*, *Tribolium castaneum*), beetle (Accession number EFA11828); (*Dappu*, *Daphnia pulex*), water flea (EFX82151.1); (*Megro*, *Megachile rotundata*), leafcutter bee (XP_003706084); and (*Bomim*, *Bombus impatiens*), bumble bee (XP_003485365).

**Figure 6:**
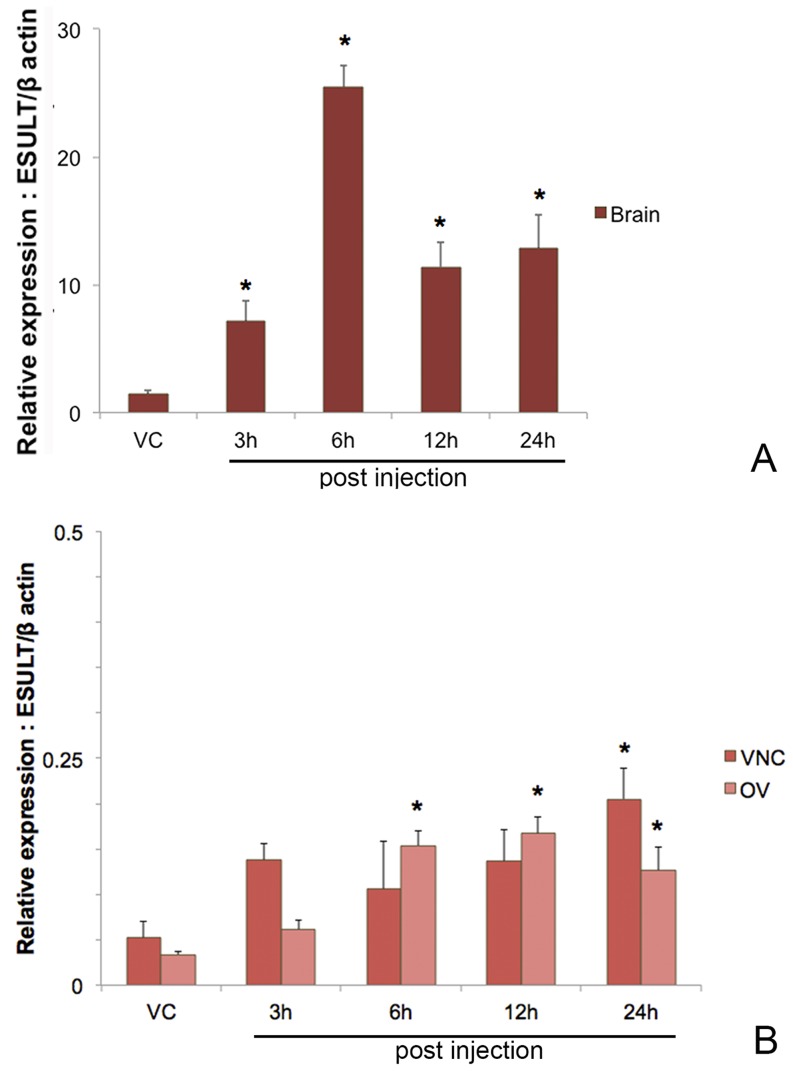
Effect of priming 5-HT on the relative *ESULT* gene expression levels of *S. olivacea* using quantitative RT-PCR. (**A**) *ESULT* mRNA expression levels in the brain (n = 12) were determined at 3, 6, 12 and 24 h post 5-HT injection. (**B**) *ESULT* mRNA expression levels in the VNC and ovary (n = 12) were determined at 3, 6, 12 and 24 h post 5-HT injection. Data were normalized against *β*-actin. Asterisks indicate significant differences (*p<*0.05) with respect to the control (vehicle-injected) group. Abbreviations: estrogen sulfotransferase (ESULT), and vehicle control (VC).

*ESULT* was identified in the *S. olivacea* transcriptome. In this study, we show the full-length protein encoded within the *Scyol-ESULT* transcript **(** **S2 Fig** **.)** and compared to deduced homolog sequences within other species, showing similarities to *D. pulex* (32%), *Tribolium castaneum* (30%), *Megachile rotundata* (29%), and *Bombus impatiens* (28%) **(** **Fig. 5** **)**. Analysis of *ESULT* protein sequence was shown for possible phosphorylation sites **(** **Fig. 5** **)**. Although estrogen, together with progesterone, are female sex steroids that play very important roles in ovarian maturation and ovulation in vertebrates, their presence in decapod crustaceans is still a subject of discussion. The presence of two sex steroids were reported in *P. monodon* [38], and *Scylla serrata* [39], but as yet, have not been found in other decapods. Here, for the first time we have unequivocally demonstrated that a critical component of the estrogen transport pathway is present and supports a role for estrogen, or an estrogen-like molecule in decapods. ESULT plays an essential role by adding a sulfate group to estradiol, so that it becomes soluble in the blood and hemolymph, enabling it to circulate throughout the body [40], [41]. Although ESULT has been well characterized in mammals its presence in invertebrate species is relatively unknown. ESULT in humans and mice is expressed in several tissues, most of which are also estrogen target sites, including the mammary gland epithelial cells, testis, and the pregnant uterus [42], [43]. Until now, ESULT has never been identified in a crustacean, even though studies have reported that estradiol occurs in the hemolymph, hepatopancreas and ovary during the gonadal maturation cycle of *S. serrata* and *P. monodon* [38], [39]. We show by qPCR the relative expression of *Scyol-ESULT* in the brain, VNC and ovary before and after 5-HT induction. In the brain, *Scyol-ESULT* expression increased significantly (*p*<0.05) following 5-HT injection at 3, 6, 12 and 24 h, compared with the vehicle control group **(** **Fig. 6A** **)**. Brain *Scyol-ESULT* levels differed from VNC and ovary *Scyol-ESULT* by more than 10 fold. *Scyol-ESULT* expression in the VNC altered after 5-HT injection only at 24 h post-injection, higher than the vehicle control group **(** **Fig. 6B** **)**. Also, *Scyol-ESULT* expression in the ovary showed a significant increase (*p*<0.05) following 5-HT injection at 6, 12 and 24 h **(** **Fig. 6B** **)**. These findings provide the first insight into a relationship between ESULT and estrogen upon 5-HT stimulation in a crustacean. The *Scyol*-*ESULT* transcript provides a potential candidate for the control of reproduction in crabs.

#### *Prostaglandin F synthase (PGFS)*

**Figure 7:**
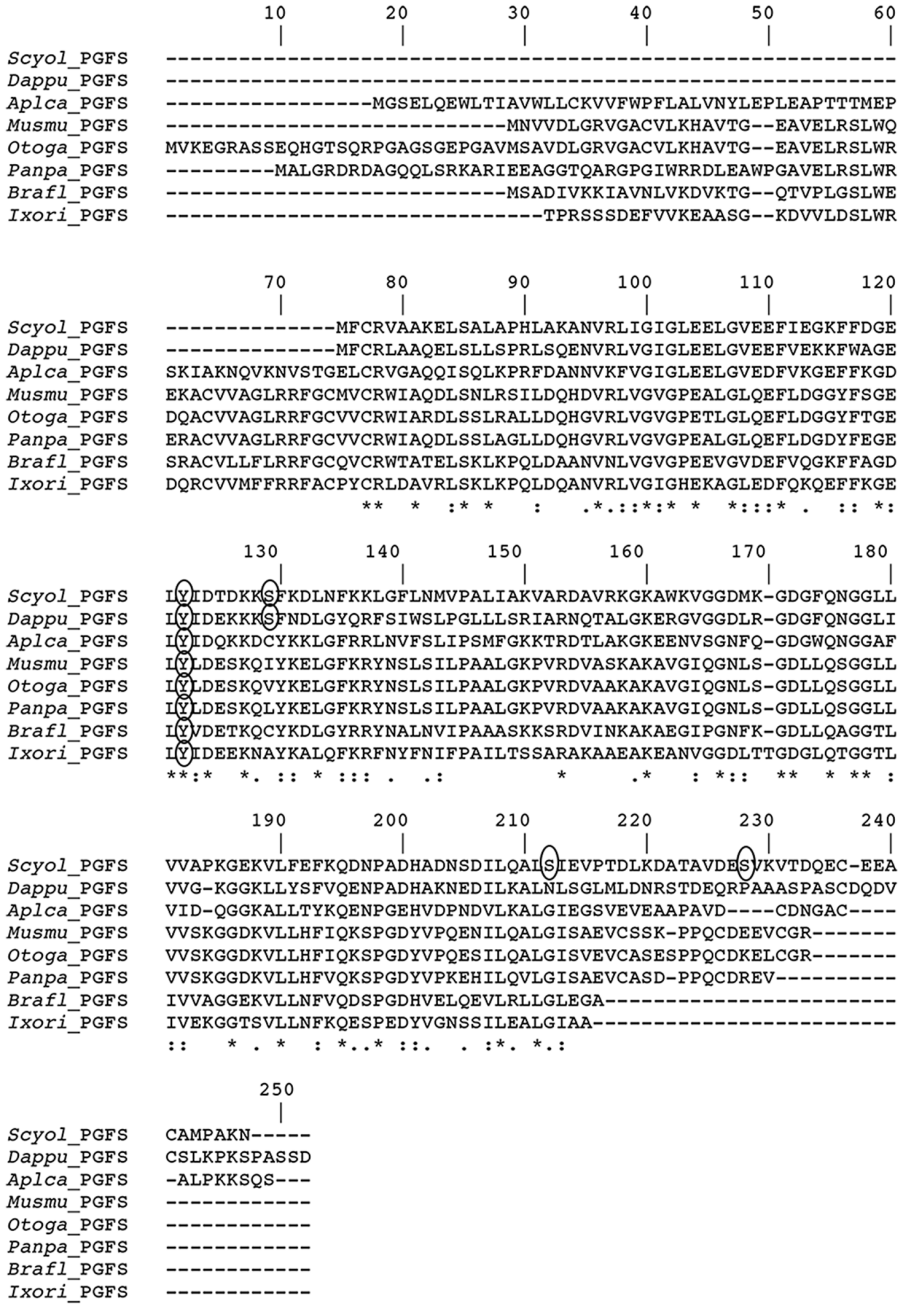
Alignment of amino acid sequence of prostaglandin F synthase (*PGFS*) of *S. olivacea* with known sequences from other species. Gaps (-) included to allow alignment. “*”: identical amino acids; “:”: conserved substitutions (same group); “.”: semi-conserved substitution (similar shapes). Circles indicating phosphorylation site prediction on protein sequences. (*Scyol*, *Scylla olivacea*), mud crab; (*Otoga*, *Otolemur garnettii*), greter galago (Accession number XP_003793364); (*Musmu*, *Mus musculus*) Mouse (NP_079858); (*Panpa*, *Pan paniscus*), bonbono (XP_003807368); (*Brafl*, *Branchiostoma floridae*), lancelet (XP_002587381); (*Ixori*, *Ixodes ricinus*), castor bean tick (JAA72765); and (*Dappu*, *Daphnia pulex*), water flea (EFX68017).

Prostaglandins (PGs), eicosanoid cell-signaling molecules derived from arachidonic fatty acids, are involved in various physiological functions in mammals, such as smooth muscle contraction [44], pain transmission [45], inflammation [45], and reproduction [46]. The presence and role of PGs have not been extensively studied in crabs [47], [48]. However, PGs have been implicated as reproductive hormones in crustaceans, especially PGF_2α_ and PGE_2_. For example, in *Penaeus japonicus*, hemolymph PGF_2α_ and PGE_2_ levels increase during stage II of ovarian developmental [49]. As well, in *Procambarus paenunsulanus*, PGs levels in ovaries gradually increase over the progression of vitellogenesis [50]. In our study, we found the enzyme responsible for PGF2α production, namely PGFS, and showed the full-length protein encoded within the *Scyol-PGFS* transcript **(** **S3 Fig** **.)**. Comparison of *S. olivacea PGFS* with those of other species shows a similarity to *D. pulex* (46%), *Branchiostoma floridae* (37%), *Mus musculus* (37%), *Ixodes ricinus* (37%), *Pan paniscus* (37%), and *Otolemur garnettii* (36%) **(** **Fig. 7** **)**. Analysis of *PGFS* protein sequence was shown for possible phosphorylation sites **(** **Fig. 7** **)**.

**Figure 8:**
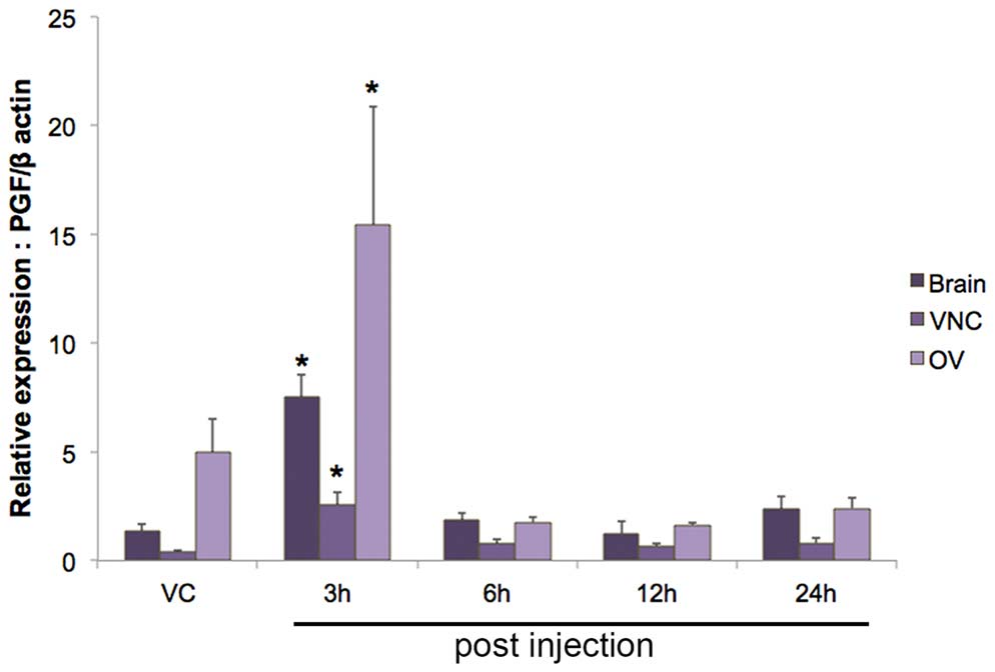
Effect of priming 5-HT on the relative *PGFS* gene expression levels of *S. olivacea* using quantitative RT-PCR. *PGFS* mRNA expression levels in the brain, VNC and ovary (n = 12) were determined at 3, 6, 12 and 24 h post 5-HT injection. Data were normalized against *β*-actin. Asterisks indicate significant differences (*p<*0.05) with respect to the control (vehicle-injected) group. Abbreviations: prostaglandin F synthase (PGFS), ventral nerve cord (VNC), ovary (OV), and vehicle control (VC).

*Scyol*-*PGFS* levels in the brain, VNC and ovary was significantly (*p*<0.05) higher at 3 h after 5-HT injection relative to those at the vehicle injection **(** **Fig. 8** **)**. The presence and expression of prostanoid biosynthesis genes have recently been reported, suggesting that they are associated with ovarian development in *P. monodon* [51]. In this study, we further indicate that 5-HT up-regulates the expression of *PGFS* mRNA, similar to other reproductive genes [7], [35]. PG isoforms, and other associate PG enzymes, will need to be identified in this species.

### Identification of putative neuropeptides with unknown functions

Approximately 495 isotigs did not show any significantly match (E-value <10^−4^) with any transcript in current databases following blast searches. To attempt to determine whether any encode putative neuropeptides, their encoded sequences were investigated through an open reading frame (ORF) prediction program [52]. Also, the presence of a putative signal peptide was predicted based on the CLC genomic workbench and SignalP 4.1 sever. Approximately 62 sequences indicated the presence of a signal peptide in their ORF suggested that they were secreted which is typical of neurohormones. Posttranslational processing of these was analysed using the Neuroprep web tool [53]. A list of putative peptides that may be released from precursors is shown in **Table 1**. Approximately 32 putative neuropeptide sequences with predicted post-translation modifications including amidation, pyroglutamination and acetylation are shown, together with their predicted theoretical ion masses. These putative neuropeptides are proposed to be cleaved from their precursor at dibasic amino acids cleavage sites. We suggest that these predicted neuropeptides might be released into an extracellular region of the CNS prior to exerting some kind of physiological regulation on cells. To determine their function, these putative peptides require further exploration into their spatial and temporal expression in *S. olivacea*, and indeed all crustacea.

**Table 1:** List of putative neuropeptides, predicted from non-hit blast search transcripts.

Contig and isotig number	Sequence (amino acid) + post-translational modification	MW (average)	Probability extracellular**
Contig 180	[p-]ESPPAFLEISTNSDFSLSLSLSLSL	2636.93	68%
Isotig 232	RPSKNPSDVTETPETSAIIGERLVSKFL[Amide]	3071.48	85.50%
	NPSDVTETPETSAIIGERLVSKFL[Amide]	2602.92	
	DWIEMSKD[Amide]	1022.14	
	DKESSPSPGEPDNSL[Amide]	1557.59	
	[p-]ESSPSPGEPDNSLG	1354.34	
	[p-]QSENTDMTAEVDTVPENPADKETEKSDKEEKEMEGEAEDDDDDDDD	10761.85	
	GNDEDRGELEDKMEEKTNMEGETQKDEGVQETKTSSSSGGAETQVSDERPQ		
	[p-]QPRMELEPETEPESKTEPEPEPEMKPEPEPKPEAEPQVATEAESTTEAT	11398.13	
	TEPTRKPEMQDESSADEQMTASETISQTETKVPAESQSDTKQNVGESSAE		
	[p-]ESSPSPGEPDNSL[Amide]	1296.3	
	[Ac-]SIAEEGTELTSKDEAAGEEGSNTDNDTTAMEDQKNTEEGNSVTDE	6306.34	
	PKNATDENKEVTGE		
	[Ac-]TTASKPGPQEVSGSLVVAGGGDSEVVVVEGVMDENQSATQG	6999.48	
	QLVMADSSLDTNTGKTHDKIHNTDETE		
	[Ac-]TEHTESDKSSEESGESIEQEDDKETTDHPSTTTDSINTTTEEDSP	7512.77	
	SIEEKQQTHIHKLLASLWQQL		
Isotig 259	[p-]ESFELTWWTQEDASCTPISTQEICLYFEQTNTQVLIFIDLWCCVGKY	5566.28	57%
Isotig 508	[Ac-]APQTNIPLHQQYVTHTDHFKYVDPKILKEIRWP	4056.63	75.40%
	[Ac-]SNSNNYGFFLPNGTTIELQHLGDGNGHQQQRNNFLSWLTERVFGIHS YI TVTAE	6166.69	
Isotig 616	[Ac-]SEEDDDNDEKSEEEDKDDDNEDEE	2916.6	91.90%
Isotig 918	[p-]QQPSNTLLAAPQLKAKIIH	2054.42	55.40%
Isotig 979	[Ac-]AAPVEVAGGEVVEPLLNLPQ	2044.33	87.20%
Isotig 1132	[p-]QQVYVRPVHGGVISQKCFIVPHFFPLSLTFSLSIKSNNPQTLLTNITGDRC	5685.6	91.40%
Isotig 1171	[Ac-]TGGKARLQSKLWTHTSVGDNIPDWF	2857.17	88.20%
Isotig 1222	[Ac-]APPPTIPLHQQYVNDGDDLKYEYPKFLRDIPSLSSNSGNYGMVLP NGTTIELQYEGDENGHQQQIIYFLPWLKDRFFEIRSHTVVVP[Amide]	10091.3	82.10%
Isotig01247	[p-]QSTTVFNVTEGDYFKFKNSVFLKGFRCLTSGSITYSIRLPNGTFARVRHITDENKY	6439.27	82.60%
Isotig 1395	[Ac-]ASYRPRN	904.98	85.10%
	[Ac-]ASYRPRNRREHPVQLDNDAGGKVLLVN	3117.47	
	[p-]EHPVQLDNDAGGKVLLVN	1900.11	
Isotig 1542	[p-]QMLGTHKTKHLKTSTPILCRIY	2553.07	68.20%
Isotig 1566	[p-]QYLCGDVICGVSCQGDGERYFFYLFFLFLTSQ	3703.21	77.60%
	[Ac-]SVRVLSIVSVVHVQMNKEFVT	2413.86	
Isotig1681	[Ac-]TTMDQATAVMAMTTNQTLDITKAMEEEVAMTTMIAGEGDTTPPK	7798.85	66.90%
	ATTMVRTEALYLDPALPSSAYRNQVRTA		
Isotig1682	[p-]QMGRIVLSFPPPTNTATSKVVVAAAPAIHSTSSCTC	8088.4	70.50%
	IHRYKAVFLTCKSQSFSQSVSQSLSLSLFLSLLPLTSTYP		
Isotig 1712	[p-]EQLIGTAIDT	1042.14	63.70%
Isotig 2049	[Ac-]SEEDDDNDEKSEEEDKDDDNEDEE	2916.6	91.90%

**Based on precursor signal sequence (SignalP 4.1).

## Conclusions

In this study, we have performed a transcriptome analysis of the CNS of *S. olivacea* following 5-HT treatment. We generated 197,468 sequence reads, comprising 2,183 isotigs and 44,721 singletons. The isotigs were annotated with 34 GO functional categories and 59 pathways. Based on our sequence analysis, we have obtained the full-length of some genes encoding proteins that are associated with reproduction, including *FAMeT, ESULT* and *PGFS*. After 5-HT injection, *FAMeT, ESULT* and *PGFS* became significantly up-regulated in the brain, VNC and ovary compared with the control group. In addition, this study reveals the existence of 32 putative neuropeptides.

## Material and Methods

### Tissue samples

Mature female *S. olivacea* were supplied by the commercial fisherman from the Gulf of Thailand, Chantaburi province, Thailand. We selected crabs with a mature stage ovary at GSI between 7 to 10. The average weight of these crabs was 400±50 g body weight and the width of their carapaces was 100–120 mm. Crabs were kept in concrete tanks, with seawater at 26–28°C and salinity at 30 ppt. Approximately 50% of seawater was changed every day, and animals were fed with commercial food pellets twice daily. The animals were acclimated under a photoperiod of 12:12 h light-dark for 1 week prior to experimentation. Wild-type mud crabs were those used for commercial consumption in Thailand. *Scylla olivacea* is not an endangered or protected species, so specific permission was not required for research project.

Injection protocols followed the method used previously [7]. The neurotransmitter serotonin (5-HT; Sigma, MO, USA), was dissolved in 0.9% normal saline and, 100 µl 5-HT (5 µg/g BW) was injected into the muscle at the base of the fifth walking leg, after which animals (n = 40) were collected at 0, 3, 6, 12 and 24 h post injection. Brains and ventral nerve cords (VNC) were dissected and immediately placed into pre chilled autoclaved 1.5 ml tubes, then quickly frozen in liquid nitrogen and stored at −80°C until use.

### Total RNA and mRNA extraction

Total RNAs were prepared from each tissue using Trizol reagent (Invitrogen, CA, USA), following the manufacturer's protocol, and kept at −80°C until use. The purity and quantity of each RNA sample was measured by a spectrophotometer at 260 and 280 nm. Extraction of mRNAs was then carried out using an Oligotex-dT mRNA Midi Kit (Qiagen, Inc., Carlsbad, CA), following the manufacturer's protocol. The mRNAs were kept at −80°C until preparation for sequencing.

### Sequencing and annotation

Pooled brain and ventral nerve cord mRNA from mature female *S. olivacea* was sent to the Australian Genome Research Facility (AGRF), Brisbane, Australia, for cDNA synthesis using a cDNA Rapid Library Preparation Kit (Roche) and subjected to 454 GS-FLX sequence analysis. After initial quality filtering, the AGRF provided assembled contig, isotig and singleton datasets for analysis.

Blastx homology searches of the GenBank non-redundant (nr) database hosted by the National Center for Biotechnology Information (NCBI) (http://www.ncbi.nlm.nih.gov/) were performed on all isotigs. All blast searches were conducted using Blast2GO software [54] with an E-value cutoff of 1e^−4^. The Blast2GO software suite was also used to predict functions of individual ESTs, assigned GO terms and their parents associated with the top 50 BLAST hits for each sequence. Moreover, Kyoto Encyclopedia of Genes and Genomes (KEGG) showed the enzymatic functions in the context of the metabolic pathways, using with an E-value cutoff of 10^−6^.

### Full-length identification of genes associated with reproduction

To identify the full-length sequences of selected genes, 3' RACE and 5' RACE were performed in female *S. olivacea* CNS using the SMART cDNA library construction kit (Clontech, CA, USA), and a 5' RACE system for rapid amplification of cDNA ends (Invitrogen, CA, USA), respectively, following the manufacturer's protocols. Universal primers provided in the kit, and gene-specific primers were used to obtain a complete gene sequence **(** **Table 2** **)**. Thermocycling conditions were: 1 cycle at 94°C for 5 min, followed by 35 cycles of 30 s at 94°C, 30 s at 52°C, and 1 min at 72°C, with a final extension of 10 min at 72°C. All amplification products were analyzed using 2% agarose gel electrophoresis. The predicted full-length size amplicon was purified using a GeneJET gel extraction kit (Thermo, USA), and cloned into a pGEM-T Easy vector (Promega, USA). Plasmids with insert sequences were purified using a GeneJET Plasmid Miniprep Kit (Thermo, USA), and then sequenced by Macrogen (Korea). Further analysis of deduced proteins from interested gene was performed with NetPhos 2.0 (http://www.cbs.dtu.dk/services/NetPhos/) on possible phosphorylation sites (score >8) at serine, threonine and tyrosine side chains within the molecules [55].

**Table 2:** Specific primers used for RACE PCR and real time quantitative RT-PCR.

Primer	Direction	Nucleotide Sequence
FAMeT-F	Forward	5′ AAGGAGTACCGCTTCAGGCAACT 3′
FAMeT-R	Reverse	5′ TATGGTCCACGGCAATCCAGAAC 3′
FAMeT-5RACER	Reverse	5′ TGGAGTTGCCTGAAGCGGTA 3′
FAMeT-3RACEF	Forward	5′ TGGAGTGGACCGACCCCGAG 3′
ESULT-F	Forward	5′ GCGTGGCAGAAGAGGCACCA3′
ESULT-R	Reverse	5′ TCCAGTCTCCCGTCTTGCCCT 3′
ESULT-5RACER1	Reverse	5′ AGGGTTGTGTAGCATGGTCCAC 3′
ESULT-5RACER2	Reverse	5′ TCACAATCACGTCGTCGCTTCTA 3′
ESULT-3RACEF1	Forward	5′ CACTTTACGCCAGAACTGCAGA 3′
ESULT-3RACEF2	Forward	5′ CATTGGATCAAAGAGAACATCGCA 3′
PGF-F	Forward	5′ GGAGGAGCTTGGCGTGGAGG 3′
PGF-R	Reverse	5′ GGGAGCCACCACAAGCAGCC 3′
PGF-5RACER1	Reverse	5′ GCCAGGTGGGGTGCCAGGGCGC 3′
PGF-5RACER2	Reverse	5′ GCGCTGAGCTCCTTGGCAGCCA 3′
PGF-3RACEF1	Forward	5′ TTGACGAGTCAGTGAAAGTAAC 3′
PGF-3RACEF2	Forward	5′ TAACAGACCAGGAATGTGAGGAG 3′
Actin-F	Forward	5′ GAGCGAGAAATCGTTCGTGACAT 3′
Actin-R	Reverse	5′ CCGATGGTGATGACCTGGCCGT 3′

### mRNA expression of genes associated with reproduction

Sixty female mud crabs, at late intermolt and with mature ovaries, were divided into five groups. The groups were: (1) injected with vehicle control with 100 µl 0.9% normal saline, and (2–5) injected with 100 µl 5-HT (5 µg/g body weight, BW) dissolved in 0.9% normal saline, into the muscle at the base of the fifth walking leg, then sacrificed at 3, 6, 12 and 24 h. The brains, VNCs and ovaries were dissected and collected from each group. The tissues were placed separately into ice-cold sterile tubes, frozen in liquid nitrogen, and stored at −80°C until use approximately 1 week later. Trizol reagent (Invitrogen, USA) was used to extract total RNA from the individual brains, VNCs and ovaries of each crab following the manufacturer's protocol. First-strand cDNA was synthesized using a Quantitect Reverse Transcription Kit (Qiagen, Germany), and then used in real-time PCR detection. Each real-time PCR was performed in duplicate for each sample. A list of gene-specific primers is provided in **Table 2**, and the primers were used for amplification of mud crab *FAMeT*, *ESULT*, *PGFS* and *β*-actin (control) genes. Amplifications (n = 12) were conducted in a 7500 Real Time PCR System (Applied biosystems, USA) using KAPA SYBR FAST qPCR kit master mix for ABI Prism (Kapabiosystems, USA). The *β*-actin gene was used as the normalization control [7]. Cycling conditions for each reaction were: 95°C for 10 min, followed by 45 cycles of 95°C for 15 s, 55°C for 10 s, and 72°C for 10 s. RotorGene's software (version 6-0-22) automatically calculated the reaction efficiencies in the reactions. The REST 2009 (Relative Expression Software Tool; Qiagen, Germany) was used to calculate relative expression against *β*-actin, before being subjected to analysis of variance between individual treatments. Statistical significance analyses were performed with a SPSS program (Statistical Product and Service Solutions; version 11), using a one-way analysis of variance (ANOVA). A probability value less than 0.05 (*p*<0.05) indicated a significant difference.

### Neuropeptide prediction

Predicted ORFs for sequences obtained from CNS transcriptome sequencing were obtained using the http://proteomics.ysu.edu/tools/OrfPredictor.html [52]. The CLC genomic workbench with a>0.68 probability value cutoff was used to predict signal peptides. Each protein was then selected for further signal peptide prediction using the SignalP 4.1 sever (http://www.cbs.dtu.dk/services/SignalP/), using a>0.5 probability value cutoff [56]. The Neuroprep processing web tool (http://neuroproteomics.scs.illinois.edu/cgi-bin/neuropred.py) [53] was used to provide predicted cleavage sites, average masses, and post-translational modifications.

## Supporting Information

S1 Fig **Full-length** ***FAMeT*** **nucleotide and deduced amino acid sequences derived from** ***S. olivacea*** **cDNA.** The amino acid sequence is shown as single letters underneath the nucleotide sequence. The first bold M represents the start methionine. The italics sequence represents the associated peptide. An asterisk indicates the stop codon. The numbers on the right indicate nucleotide and amino acid numbers. *FAMeT* deduced protein consisted of two domains, showing in green and orange. Circles indicated phosphorylation site prediction on deduced protein.
(TIF)

S2 Fig **Full-length** ***ESULT*** **nucleotide and deduced amino acid sequences derived from** ***S. olivacea*** **cDNA.** The amino acid sequence is shown as single letters underneath the nucleotide sequence. The first bold M represents the start methionine. The italics sequence represents the associated peptide. An asterisk indicates the stop codon. The numbers on the right indicate nucleotide and amino acid numbers. Circles indicated phosphorylation site prediction on deduced protein.
(TIF)

S3 Fig **Full-length** ***PGFS*** **nucleotide and deduced amino acid sequences derived from** ***S. olivacea*** **cDNA.** The amino acid sequence is shown as single letters underneath the nucleotide sequence. The first bold M represents the start methionine. The italics sequence represents the associated peptide. An asterisk indicates the stop codon. The numbers on the right indicate nucleotide and amino acid numbers. Circles indicated phosphorylation site prediction on deduced protein.
(TIF)

S1 Table **The representation of full-length ORF of transcriptome data.**
(XLSX)

S2 Table **KEGG analysis of transcriptome data.**
(XLSX)
