# Supplementary figures and images for: Identification of Genes Associated with Reproduction in the Mud Crab (Scylla olivacea) and Their Differential Expression following Serotonin Stimulation
Source: PLoS One. 2014 Dec 26;9(12):e115867. doi: 10.1371/journal.pone.0115867 (PMC4277393; doi:10.1371/journal.pone.0115867)

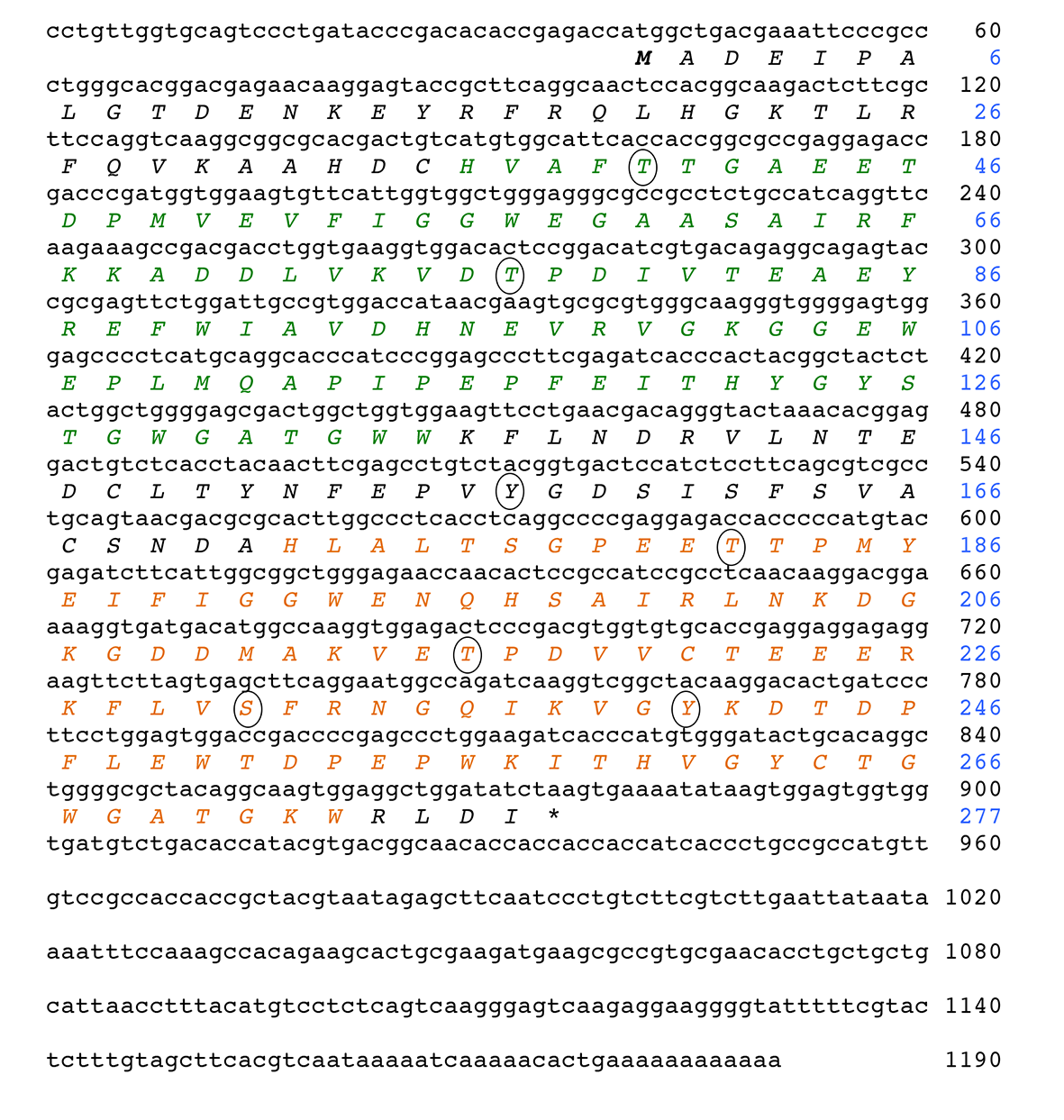

Supplement: S1 Fig — Full-length FAMeT nucleotide and deduced amino acid sequences derived from S. olivacea cDNA. The amino acid sequence is shown as single letters underneath the nucleotide sequence. The first bold M represents the start methionine. The italics sequence represents the associated peptide. An asterisk indicates the stop codon. The numbers on the right indicate nucleotide and amino acid numbers. FAMeT deduced protein consisted of two domains, showing in green and orange. Circles indicated phosphorylation site prediction on deduced protein. (TIF) [file pone.0115867.s001.tif]

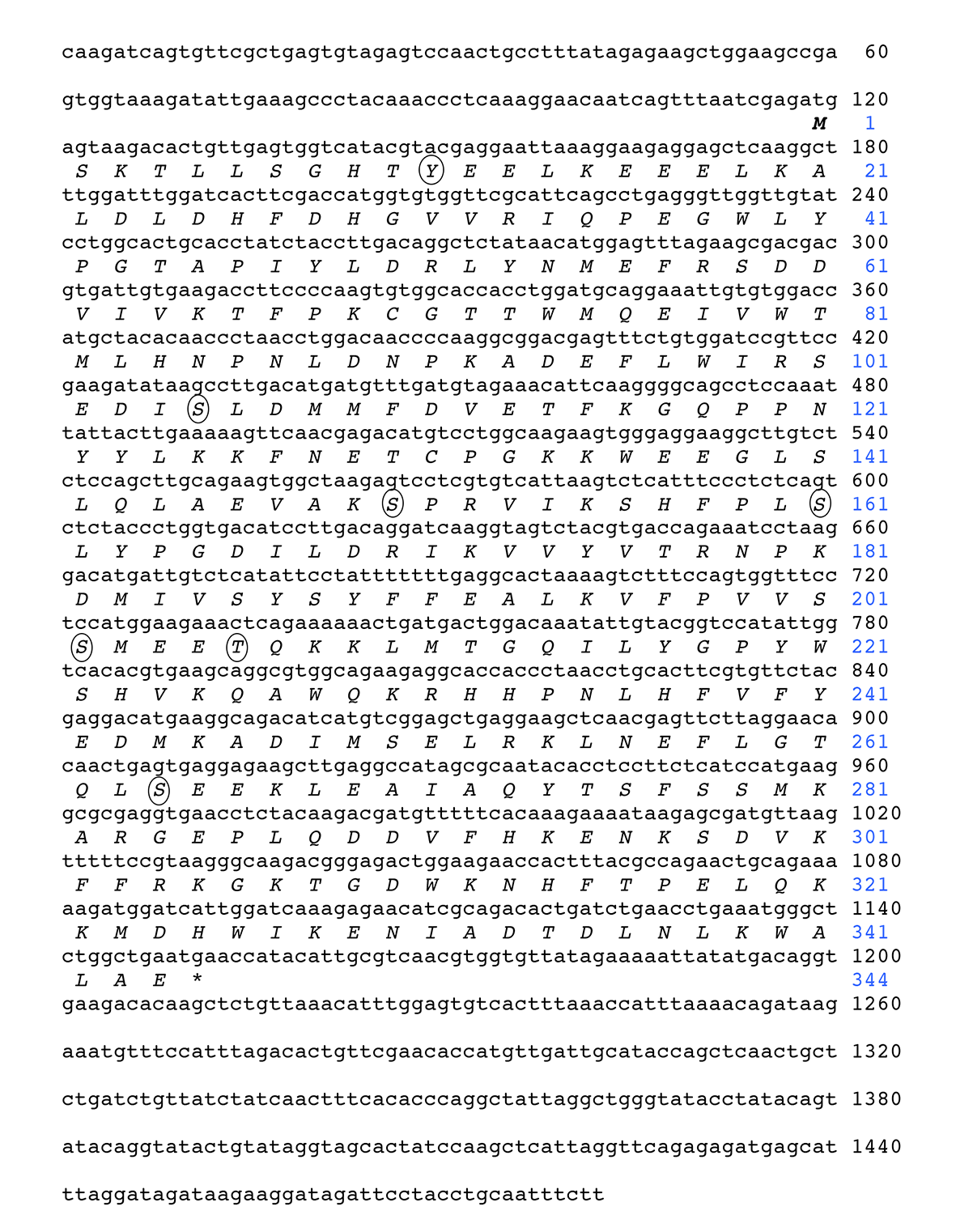

Supplement: S2 Fig — Full-length ESULT nucleotide and deduced amino acid sequences derived from S. olivacea cDNA. The amino acid sequence is shown as single letters underneath the nucleotide sequence. The first bold M represents the start methionine. The italics sequence represents the associated peptide. An asterisk indicates the stop codon. The numbers on the right indicate nucleotide and amino acid numbers. Circles indicated phosphorylation site prediction on deduced protein. (TIF) [file pone.0115867.s002.tif]

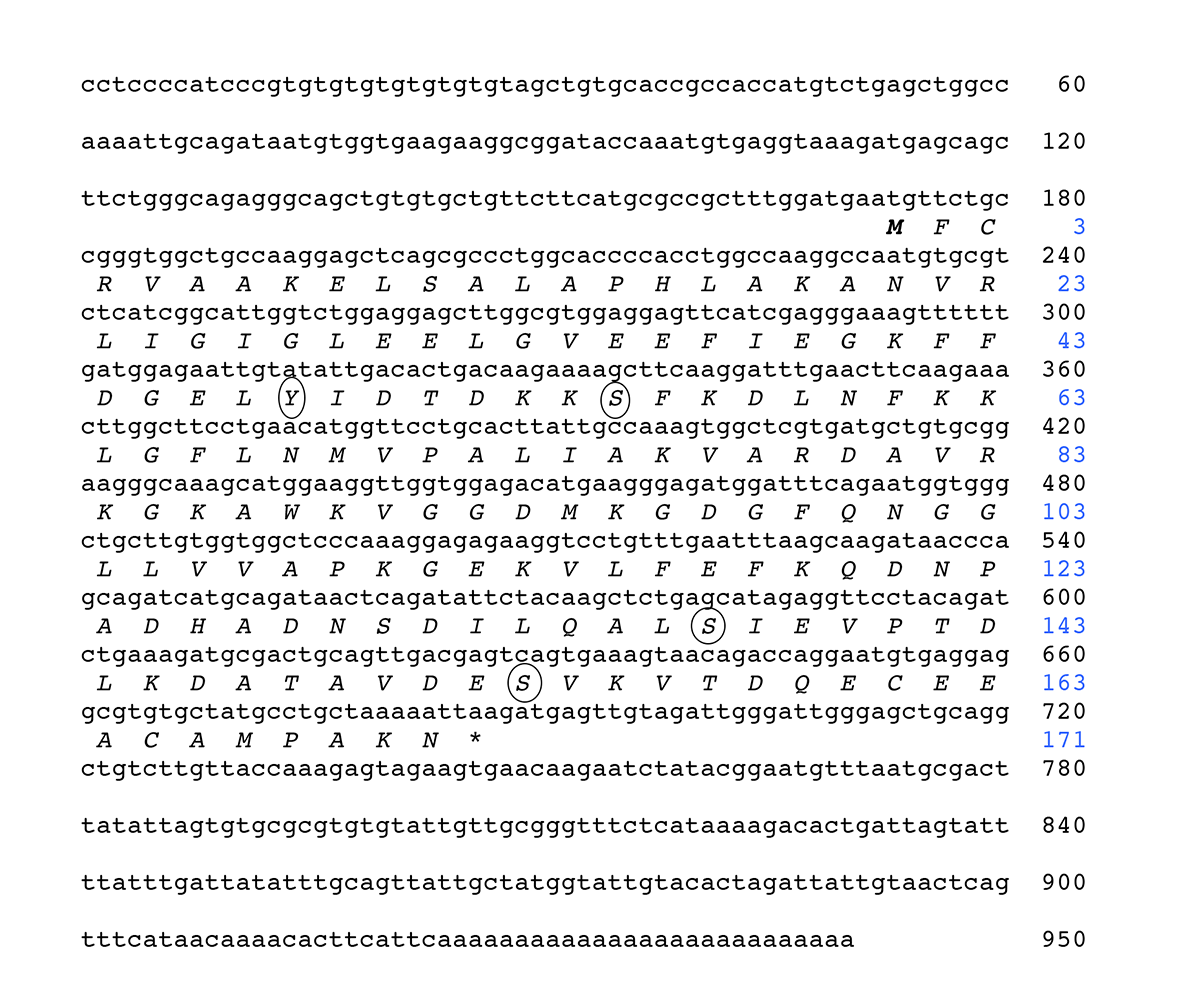

Supplement: S3 Fig — Full-length PGFS nucleotide and deduced amino acid sequences derived from S. olivacea cDNA. The amino acid sequence is shown as single letters underneath the nucleotide sequence. The first bold M represents the start methionine. The italics sequence represents the associated peptide. An asterisk indicates the stop codon. The numbers on the right indicate nucleotide and amino acid numbers. Circles indicated phosphorylation site prediction on deduced protein. (TIF) [file pone.0115867.s003.tif]
